# Innate Immune Responses in House Dust Mite Allergy

**DOI:** 10.1155/2013/735031

**Published:** 2013-02-28

**Authors:** Alain Jacquet

**Affiliations:** Division of Allergy and Clinical Immunology, Department of Medicine, Faculty of Medicine, Chulalongkorn University, Oor-Por-Ror Building, 10th Floor, Room No. 1010/5, 1873 Rama IV Road, Pathumwan, Bangkok 10330, Thailand

## Abstract

Sensitizations to house dust mites (HDM) trigger strong exacerbated allergen-induced inflammation of the skin and airways mucosa from atopic subjects resulting in atopic dermatitis as well as allergic rhinitis and asthma. Initially, the Th2-biased HDM allergic response was considered to be mediated only by allergen B- and T-cell epitopes to promote allergen-specific IgE production as well as IL-4, IL-5, and IL-13 to recruit inflammatory cells. But this general molecular model of HDM allergenicity must be revisited as a growing literature suggests that stimulations of innate immune activation pathways by HDM allergens offer new answers to the following question: what makes an HDM allergen an allergen? Indeed, HDM is a carrier not only for allergenic proteins but also microbial adjuvant compounds, both of which are able to stimulate innate signaling pathways leading to allergy. This paper will describe the multiple ways used by HDM allergens together with microbial compounds to control the initiation of the allergic response through engagement of innate immunity.

## 1. Introduction

House dust mites (HDM; *Dermatophagoides sp.*) are one of the commonest sources of airborne allergens worldwide and we can consider that HDM sensitization affects more than 15–20% of the population from industrialized countries [1]. Atopic patients exposed to HDM allergens develop potent inflammatory diseases in such allergic asthma, perennial rhinitis, and atopic dermatitis (AD) [2].

Experimental evidences suggest that HDM allergen-specific Th2 cells play the central role in the allergic inflammatory response inducing the production of allergen-specific IgE, the eosinophil recruitment in tissues, the permissiveness of endothelium for the recruitment of inflammatory cells to inflamed lungs, the production of mucus, and the modulation of the airway smooth muscle contraction. Notably, the Th2 cytokines IL-4, IL-5, IL-13 orchestrate these inflammatory processes: IL-4 is important for allergic sensitization and IgE production, eosinophil survival depends mainly on IL-5, whereas IL-13 has pleiotropic effects in the lungs, including a central role in the development of AHR and tissue remodeling [3].

Despite the high prevalence of HDM allergy, the precise nature of the cellular and molecular networks that initiate and regulate this Th2-biased response is still unclear. Recent advances clearly demonstrated that the HDM allergic response can no longer be considered as a unique dys-regulation of the adaptive immune system. Actually, a crosstalk between the innate and adaptive immune system plays a critical role in the initiation and propagation of the allergic Th2 response. Recent reports highlighted that innate immune cells including bronchial epithelial cells and keratinocytes can indeed be directly activated by both the HDM allergens themselves and danger signals present in the HDM body or in the environment [4].

This paper will describe the diverse determinants that contribute to HDM allergenicity through the activation of innate immunity and will reflect how these HDM-induced innate immune responses are critical for the development of the Th2-biased adaptive immunity to HDM allergens. 

## 2. New Insights into the Mechanism of the Allergen Sensitization

It is well known that dendritic cells (DCs) have a pivotal role in the activation of Th2 cells and allergic inflammation. DCs are present in the tissues of the body exposed to the exterior environment such as the skin and the epithelia of the lung and gut [5]. They play the role of sentinels to sense the tissues for incoming antigens. DCs express a large set of receptors of the innate immune system, the pathogen recognition receptors (PRRs), and also have the potential to take up allergens, to process these proteins into small peptides, and then to present them through the major histocompatibility complexes MHC class I and MHC class II for recognition by T-cell receptors. Initially, the typical model of allergen sensitization could be described as a multistep process consisting in (a) the uptake of the inhaled allergen by DCs, a process which can be facilitated by interaction of the allergen with IgE attached to Fc*ε*RI, the high-affinity receptor for IgE, present on DCs, (b) the PRRs activation on the surface of the DCs, (c) the migration and maturation of the DCs from the peripheral tissues to the draining lymph nodes, and (d) the allergen presentation to the allergen-specific Th cells together with the Th2 differentiation.

The differentiation of activated naive T cells into functional T-cell subsets (Th1, Th2, Th17, Treg at least) depends largely on the DC-derived cytokines patterns [6]. Although the signals needed to drive Th1 and Th17 polarization by DCs are now well characterized (IL-12 and IL-6/TGF-*β* for Th1 and Th17 differentiation, resp.), the pathway of the Th2 differentiation remained to be fully elucidated. Although it is clearly evident that the Th2 polarization requires a source of IL-4 to activate the transcription factors STAT6 and GATA3, DCs were found unable to secrete IL-4. The inability of DCs to produce IL-4 leads to two hypotheses explaining the Th2 polarization: Th2 differentiation could be considered as a default state in the absence of strong Th1 or Th17 cytokine milieu in the DC-T synapse or the IL-4 source is from other innate immune cells.

It has long been known that basophils produce large amounts of IL-4 when activated by Fc*ε*RI cross-linking or through other cell surface receptors [7]. Recent papers showed that basophils transiently enter lymph nodes during immune responses to papain and that they are essential for papain-specific IgE production and Th2 cell responses in mice. Basophils were acting as typical antigen presenting cells through the uptake and the processing of papain, the costimulatory molecules expression, and IL-4 secretion [8]. However, the basophil-depleting antibody used to evidence the critical role of basophils in the development of Th2 immunity also was able to deplete a subpopulation of inflammatory DCs [9]. More strikingly, Th2 response induced by HDM was only partially reduced in the absence of basophils, and basophils were not able to present HDM allergens to Th cells to drive the Th2 polarization. In contrast, the specific depletion of CD11c^hi^ DCs in CD11c-DTR Tg clearly demonstrated that DCs alone are sufficient for the specific HDM allergen-specific Th2 polarization [9]. Consequently, basophils could not be considered as the initiators but the amplifiers of the Th2 immunity to HDM.

In recent years it has become clear that airway epithelial cells, the first cell type to be exposed to inhaled airborne allergens, cannot be only considered to display passive barrier functions, but also actively contribute to the induction of the allergic response [10]. Epithelial cells express a large variety of PRRs to respond to microbial pathogen-associated molecular patterns (PAMPs) or to damage-associated molecular patterns (DAMPs) released upon tissue damage, cell death, or cellular stress. Recent studies evidenced that prolonged epithelial PRRs activation by the allergens themselves or by microbial contaminating PAMPs represent one of the key steps in the Th2 cell sensitization process which results in the release of large amounts of proinflammatory cytokines, chemokines, growth factors amplifying the influx of Th2 cells, DCs, eosinophils, basophils, and other inflammatory cells [11]. 

In particular, the release of GM-CSF, IL-25, IL-33, and TSLP from the airway epithelial cells following such activations induces the recruitment and the activation of DCs to promote Th2-biased airway inflammation through the inhibition of IL-12, the induction of chemokines as MDC/TARC which attract Th2 cells, or the overexpression of OX40L at the DC surface that can instruct Th2 cell development [12, 13]. 

Epithelial-derived chemokines/cytokines also activate innate immune cells such as basophils, mast cells, and eosinophils to sustain the Th2 priming. The recent identification of heterogeneous populations of innate lymphoid cells (ILCs), the missing link between innate and adaptive immunity, constituted an important breakthrough in the understanding of the initiation of the allergic response. Indeed, ILCs, including nuocytes, and innate type 2 helper cells can be stimulated by IL-25 and IL-33 produced by epithelial cells to become an important source of IL-5 and IL-13 [14].

Taken together, it becomes evident that the initiation of the Th2-biased allergic response results from the complex interconnection of several molecular pathways involving adaptive and innate immunity. However, the full elucidation of the molecular networks that orchestrate Th2 responses in humans must arise from a better understanding of the role of epithelial-derived cytokines and ILCs.

## 3. PRRs, Inflammation, and Airway Epithelial Cells

Innate immunity activation has been extensively characterized. At least four classes of PRRs identified to date are able to sense microbial infection to subsequently stimulate proinflammatory responses [15]. These include Toll-like receptors (TLRs), located at the cell surface or in endosomes; NOD-like receptors (NLRs), present in the cytoplasm; RIG-I-like receptors (RLRs), also located intracellularly; C-type lectin receptors (CLRs), cell surface receptors recognizing carbohydrate structures. These PRRs are expressed not only in antigen-presenting cells (macrophages, DCs) or inflammatory cells (eosinophils, basophils), but also in various nonprofessional immune cells as keratinocytes, epithelial cells, endothelial cells and fibroblasts. The PAMPs recognition by cellular PRRs trigger downstream signaling pathways, such as the NF*κ*B, MAPK and type I interferon pathways to induce the production of proinflammatory cytokines and chemokines to initiate the inflammatory and antimicrobial responses. 

But it appeared clear that PRRs also recognize endogenous danger signals, molecules released during cellular injury, in the absence of any microbial infection (sterile inflammation) [16]. These DAMPs (danger-associated molecular patterns) such as heat shock proteins (HSPs), ATP, and uric acid, like the PAMPs, have the capacity to activate proinflammatory signaling pathways. Although, the precise molecular mechanisms by which sterile inflammation occurs remain to be fully elucidated, some inflammasomes such as NLRP3, intracellular protein complexes containing PRR, were shown to sense nonmicrobial molecules [16]. Interestingly, it has been suggested that exposure to low levels of PAMPs and DAMPs is responsible for priming the Th2 immune response. Such PRRs stimulations lead to activation of APCs to trigger antigen-specific Th2-biased adaptive immune responses. As we will describe later in this paper, numerous signaling pathways induce the Th2 polarization through the preparation of a pro-Th2 cytokine milieu. One typical example is the Th2 bias induced by the alum adjuvant. Alum was shown to program DCs to induce Th2 immune response through the uric acid-dependent activation of NLRP3 inflammasome [16].

It must be pointed out that airway epithelial cells express a large panel of PRRs including TLR2-5, NOD1-2, NLRP3, Dectin-1, and PAR-2 to rapidly detect and respond to microbial PAMPs and DAMPs released upon tissue damage [17]. In the context of allergy, the stimulation of the airway epithelium by the inhaled allergens associated with the contaminating microbial compounds influences strongly the function of DCs present in tissues underneath the epithelium layer. Such crosstalk between epithelial cells and DCs is a key event to instruct DCs to promote Th2 sensitization.

## 4. HDM Allergens

The mites are members of the subclass Acari and the taxonomy roughly classified mites into the *Pyroglyphidae *family or in other families collectively called storage mites. The most predominant HDM isolated from dust samples are *Dermatophagoides pteronyssinus *and *Dermatophagoides farinae*. Both coexist worldwide although *D. pteronyssinus* prevail much more in UK, Australia, and New Zealand [18]. It must be pointed out that this paper will be focused uniquely on *D. farinae* and *D. pteronyssinus*.

To date, we can consider that more than twenty different allergens which elicited specific IgE responses in sensitized patients were identified in *D. farinae* and *D. pteronyssinus* [19]. These HDM allergens were classified according to a Linnean system of nomenclature that is maintained by the World Health Organization (WHO) and the International Union of Immunological Societies (IUIS) Allergen Nomenclature Sub-Committee. HDM allergens are named Der (the first three letters of the *Dermatophagoides* genus), p or f (the first letter of the *pteronyssinus* or *farinae* species), and a number representing the order in which they were purified or classified [20]. It is very well known that allergens from *D. pteronyssinus* and *D*. *farinae *display sequence homologies as well as similar biological activities and consequently constitute allergen groups encompassing not only the *pteronyssinus* and *farinae* species but also the *siboney* as well as the *microceras* species, together with members from other genus as *Blomia *or *Euroglyphus*.

**Figure 1:**
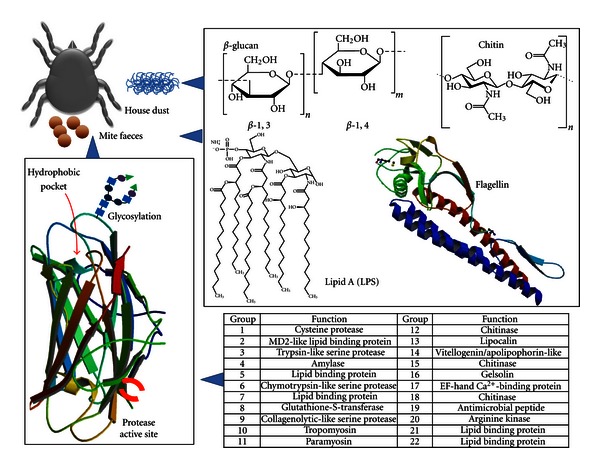
Allergenic determinants associated to house dust mite. The identified HDM allergens present in mite bodies and faeces display, together with IgE-binding and T-cell epitopes, at least one allergenic determinant able to trigger innate immunity: glycan structure to engage CLRs, hydrophobic pocket to transport lipids which, in turn, can stimulate TLRs, and protease activity to activate PAR-2 and more likely other cell surface receptors. HDM allergens are commonly associated with microbial endosymbiotic PAMPs from the house dust including at least LPS, *β*-glucans, chitin, and flagellin (crystal structure of cell-surface flagellin 2ZBI from RCSB PDB).

Although the biological function of HDM allergens in the mites remain to be fully elucidated, we can categorize these allergenic proteins into four main families: proteases, proteins displaying affinities for lipids, non proteolytic enzymes, and non enzymatic components (Figure 1) [21]. It must be pointed out that, depending on the allergen concentration in the mite, such identification and characterization of the different HDM allergen groups were allowed through the use of the natural version of the proteins or through cDNA cloning and recombinant protein productions. Group 1 HDM allergen was considered as a papain-like cysteine proteinase whereas groups 3, 6, 9 are trypsin-like, chymotrypsin-like, and collagenolytic-like serine proteinases, respectively. These proteinases could more likely play a digestive function for the mite as they were detected in the gut as well as the mite faeces [22]. According to their structural/sequence homologies, groups 2, 5, 7, 13, 14, and 21 could be described as fatty acid/lipid binding proteins but their role in the mite is still unknown [23]. Other HDM allergens display enzymatic activities: group 4, 8 and 20 allergens are amylases, glutathione-S-transferases, and arginine kinases, respectively, whereas groups 12, 15, and 18 display homologies with chitinases [24–27]. The muscle-derived proteins tropomyosin and paramyosin constitute the HDM allergen groups 10 and 11, respectively [28, 29]. Groups 16 and 17 were identified as gelsolin-like and EF-hand Ca^2+^-binding proteins [30, 31].

## 5. HDM: A Complex Combination of Stimulators Triggering Innate Immunity 

Although the HDM allergens are present in the mite bodies, the main allergenic sources are the mite faeces which, with a diameter higher than 10 *μ*m [32], can be easily inhaled into the airways and consequently be entered deep into the lungs [33]. Whereas the full composition of mite faeces remains to be determined, we can easily speculate that mite faeces contain not only the identified HDM allergens but also numerous non allergenic mite proteins and macromolecules. In the context of innate immunity activation, all these protein and non protein compounds could putatively participate to those stimulations. 

Consequently, we cannot consider HDM strictly as an allergen carrier but also as an important transporter of microbial PAMPs able to trigger innate immunity. At least three different microbial PAMPs can be detected routinely in the mite faeces and/or in the mite environment: LPS, *β*-glucan, and chitin (Figure 1). House dust, the mites' natural home environment contains large amounts of LPS and/or bacteria as well as *β*-glucans and/or fungi which can be associated with HDM [34]. Moreover, chitin, a glucosamine-based polymer not only present in the fungi cell wall but also in the mite exoskeleton, has been found in the faeces. 

It is noteworthy that a lot of studies (described later in the paper) evaluate the innate immunity activation by HDM allergens through the use of allergen extracts prepared from mite cultures, as the allergen concentration was too low starting from dust collections. The use of HDM extracts raised at least two important questions: the adjuvant factors present in the HDM extracts refer to microbial contaminations or really endosymbionts-derived molecules? The microbial compounds found in HDM cultures are the same than those present in the mite environment (house dust)? Bacterial 16S ribosomal DNA was identified in washed HDM suggesting the presence of endosymbionts in these mites [35], The microbial compounds LPS and *β*-glucans can be routinely detected in HDM extracts obtained from whole mite cultures or mite bodies [34]. Yeast enters frequently in the composition of mite growth medium and consequently could interfere with the *β*-glucan and/or chitin composition of the allergen extracts [36]. Finally, it should be pointed out that commercial HDM extracts can largely vary according to their preparation, resulting in markedly different biochemical properties such as endotoxin content [37].

The contribution of the intrinsic biological properties of HDM allergens to their allergenicity remains pending for numerous groups. To fully characterize the allergenic determinants of each HDM allergens, one method could consist in the replacement of HDM extracts by the individual purified proteins. Purification of natural HDM allergens was finally found to be very limited according to the allergen content in HDM cultures. Surprisingly, although a large panel of HDM allergens has become available to analyze the allergenicity of these proteins through recombinant DNA molecular techniques [38], the full elucidation of the allergenic determinants in the different HDM allergen groups remained elusive. During the last decade, we can consider that the stimulation of the innate immunity by HDM allergens to skew the immune response to Th2 was only demonstrated for four HDM allergen groups: the protease allergens groups 1, 3, 6, and 9 as well as the lipid-associated group 2 proteins. The next sections of the present paper will describe in detail the activation of innate immunity through these HDM allergens and adjuvant factors present in the HDM extracts.

## 6. TLR Activation in HDM Allergy 

The TLR4 ligand LPS from Gram-negative bacteria was certainly the most studied danger signal in the context of the airway allergic inflammation. As discussed above, endotoxins are the most important microbial products which contaminate HDM allergens. Allergen sensitization and development of atopic asthma were shown to be inversely related to endotoxin levels in house dust [39, 40]. Interestingly, polymorphisms of the genes encoding TLR4 and CD14 were found to influence the severity of asthma and its relation with endotoxin exposure [41–43].

Although epidemiological studies and mouse models gave sometimes contradictory results, animal models provided mechanistic insight into the role of LPS in the regulation of allergic asthma. In relation with the hygiene hypothesis, it appeared that the LPS dose is a determining factor in the course of allergic responses: low doses of inhaled LPS promoted Th2 responses to the sensitizing antigen and eosinophilic inflammation, whereas high doses of LPS induced protective Th1 responses through downregulation of Th2-type cytokines, prevention of eosinophilic inflammation, and airway hyperresponsiveness but induction of neutrophil recruitment [44, 45]. 

In an intranasal HDM sensitization mouse model, common parameters of airway allergy caused by HDM exposure as airway inflammation, Th2 cytokine production, and airway hyperreactivity were strongly attenuated in TLR4(−/−) or MyD88(−/−) mice compared with those in wild-type mice [46]. Such prevention of the allergen-specific Th2 response was associated with fewer OX40L-expressing myeloid DCs in the draining lymph nodes during allergic sensitization. HDM-specific IL-17 production and airway neutrophilia were attenuated in MyD88−/− but not TLR4−/− mice. These data suggested that the presence of microbial products in HDM extracts, more likely LPS, differentially regulates Th2- and Th17-mediated inflammation and activates distinct MyD88-dependent pattern recognition receptors. As DCs express TLR4 and considering the crucial role of these cells in the initiation and maintenance of adaptive Th2 responses in asthma, it was originally considered that the direct TLR4 activation of DCs is the key event to initiate the Th2 polarization.

However, recent experiments conducted on mice with selective ablation of TLR4 expression on either lung structural cells or hematopoietic cells clearly highlighted that the recruitment and activation of subepithelial DCs in response to LPS were controlled only by the epithelial TLR4 activation [47]. When the same animals were treated with HDM extracts, containing 1ng LPS per mg extract, only TLR4 expression on lung structural cells, but not on DCs, was necessary and sufficient for DC activation in the lung and for the development of a robust eosinophilic and Th2 inflammatory response characterized by IL-5 and IL-13 production. 

The TLR4 triggering on epithelial cells by contaminating LPS from HDM extracts promoted secretion of the innate pro-Th2 cytokines TSLP, GM-CSF, IL-25, and IL-33. Finally, inhalation of a TLR4 antagonist to target exposed epithelial cells suppressed the features of asthma, including bronchial hyperreactivity. These findings gave epithelial cells a pivotal position in the generation of allergic inflammation through the activation of TLR4 signaling pathway by the contaminating LPS from HDM.

Very recent and complementary experiments using the same HDM allergy mouse model elucidated the mechanism leading to GM-CSF and IL-33 following TLR4 signaling in bronchial epithelial cells [48]. The TLR4 activation triggered the release of IL-1*α* which, in turn, stimulated in an autocrine manner the epithelium to induce the secretion of DC-attracting chemokines, GM-CSF, and IL-33. 

A recent report confirmed the importance of TLR in the development of HDM allergy through the absence of the common features of allergic asthma in IRF3-deficient mice [49]. IRF3, a transcription factor leading notably to type I interferon gene expression, is activated by TLR3-4, 7–9. 

It is interesting to note that different results were obtained from *in vitro* experiments using differentiated primary cultures of human airway epithelia or airway epithelial cell lines. Such cells were shown to be largely hyporesponsive to LPS in comparison with phagocytic cells [50, 51]. This hyporesponsiveness could be explained by the intracellular TLR4 expression in pulmonary epithelial cells lines [50]. But, more interestingly, a deficiency of MD-2 expression, the LPS-binding accessory TLR4 coreceptor, was observed in the differentiated primary culture of epithelia and extracellular complementation with recombinant MD-2/LPS increased endotoxin responsiveness [51].

Strikingly, Der p 2 was shown to display structural homology with MD-2 [52]. This MD-2 molecular mimicry could drive allergic airway inflammation in a TLR4-dependent manner under conditions of very low levels of LPS exposure [52]. A Der p 2 + LPS airway sensitization model triggered experimental allergic asthma in wild type and MD-2-deficient, but not TLR4-deficient mice, clearly confirming that Der p 2 can transport LPS to TLR4. Consequently, the allergenicity of group 2 mite allergens results from these auto-adjuvant properties and the LPS binding activity of Der f 2 was also recently evidenced [53]. Because of the presence of hydrophobic pocket into the structure of group 2 HDM allergens, such proteins could also bind other lipids than LPS which could potentially activate TLR1/TLR2 and TLR2/TLR6 heterodimers displaying affinity for bacterial lipopeptides/lipoproteins. Recombinant Der p 2 was indeed able to stimulate airway smooth muscle cells in a TLR4-independent manner but triggered the MyD88 signaling pathway through TLR2 [54]. Der p 2 was also able to trigger human B-cell activation through NFkB-dependent IL-1*β*, CXCL10, IL-8, and TNF-*α* as well as TLR4/MD-2 induction [55].

With the exception of the TLR2/4 activation by group 2 HDM allergens, the putative association of other mite allergens with lipids remains to be identified. Elucidation of the radiocrystallographic structure of Der p 5 demonstrated the presence of a large hydrophobic pocket when this allergen dimerized. This cavity could hence represent a hydrophobic ligand binding site allowing, similarly to group 2 HDM allergens, to transport lipid-based PAMPs [56]. It must be pointed out that Der p 5 stimulated the production of IL-6 and IL-8 in human airway epithelial cells although the activation mechanism remains to be elucidated [57]. No studies evaluated to date the direct stimulation of innate immune cells by group 7 allergens but this group exhibits structure similarities with the LPS-binding protein (LBP) [58, 59]. Group 7 mite allergens, contrary to group 2, did not bind LPS but can interact with others lipid including the bacterial lipopeptide polymyxin B. It is noteworthy that HDM allergen groups 13, 14, and 21 can, according to sequence homology, also be considered as lipid-binding proteins [23]. All these lipid carriers could facilitate the allergen sensitization through putative activation of PRRs such as TLR2. 

The recent contribution of flagellin, a bacterial protein present in house dust, in the priming of the allergic response strengthened the hypothesis on the role of microbial contaminating adjuvant in HDM allergy, although future in-depth studies must address the relationships between HDM allergy and TLR5 signaling. House dust flagellin promoted the development of allergic asthma by TLR5-dependent priming of allergic responses to inhaled ovalbumin [60]. Moreover, this TLR5 ligand was shown to stimulate the *in vitro* production of TSLP in airway epithelial cells as well as in keratinocytes [61, 62].

Finally, HDM-derived *β*-glucans, rather than LPS, were described as the main PAMPs for the TLR2-dependent activation of the innate immunity in nasal mucosa and the triggering of allergic rhinitis. In contrast, LPS, rather than *β*-glucans, was critical for HDM-induced TLR4-dependent innate immunity in lung mucosa and allergic asthma. Moreover, both *β*-glucans and LPS mediated innate immunity through reactive oxygen species (ROS) production. This tissue-specific activation by *β*-glucans and LPS remains to be explained because comparable TLR2 and TLR4 cell surface expression was observed in nasal and bronchial epithelial cells [63].

## 7. C-Type Lectin Activation in HDM Allergy

HDM extracts were shown to induce CCL20 secretion in airway epithelial cells for the recruitment of immature DCs to the lung [64] through a TLR-independent, protease-independent process. The CCL20 production was drastically reduced following the treatment of HDM extracts with *β*-glucanase or the competition with *β*-glucans. Consequently, CCL20 secretion could be more likely mediated by ligation of HDM-derived *β*-glucans to the C-type lectin receptor Dectins. Whether Dectin-1 is expressed predominantly on the surface of myeloid lineage cells, it is noteworthy that some papers reported that Dectin-1 expression is inducible in airway epithelium incubated with fungal allergens [65]. Such upregulation of Dectin-1 expression was not evaluated to date for HDM-activated airway epithelial cells. The importance of CLR activation in the HDM allergic responses was also supported by data showing that DCs treated with HDM extracts produced cysteinyl leukotrienes (Cys-LTs) through Dectin-2 activation by unidentified glycan-derived molecules [66]. Moreover, Dectin-2 is critical for the development of HDM-induced influx of eosinophils, neutrophils into the lungs, and the generation of Th2 cytokines [67].

Glycan structures of Der p 1 and Der p 2 were shown to promote the uptake of these allergens by DCs following binding with mannose receptor and DC-SIGN [68, 69]. The mannose receptor engagement played a key role in the Th2 polarization through modulation of indoleamine 2,3-dioxygenase (IDO) activity. Binding of the naturally glycosylated Der p 2 to DC-SIGN on DCs elicited the release of TNF-*α*, whereas the unglycosylated recombinant form was ineffective in the receptor activation [70]. The production of TSLP in the bronchial epithelial cell lines BEAS-2B activated by natural Der p 1 was clearly dependent on the presence of the allergen glycosylation [71]. It was also showed that monocyte-derived DCs (MDDCs) from HDM-sensitized asthmatics exhibited decreased expression of DC-SIGN, increased endocytosis, and impaired differentiation of DC precursors compared with MDDCs from healthy subjects [72]. DC/T co-culture experiments using MDDCs pretreated with Der p induced GATA-3 expression and IL-4 production in naive Th cells. These effects of Der p on the differentiation and function of MDDCs could be partially blocked by anti-DC-SIGN antibodies suggesting that DC-SIGN could play a critical role in HDM sensitization [72].

Chitin, a polymer of *β*-(1–4)-poly-N-acetyl-D-glucosamine repeating units, constitutes the HDM exoskeleton and was also found in the mite faeces. Chitin, through stimulation of at least TLR2, Dectin-1 or mannose receptor, was able to polarize Th1, Th2, and Th17 immune responses [73]. This immunomodulation was clearly dependent on the size of the chitin polymers. The *in vivo* chitin administration was shown to recruit IL-4-positive innate immune cells, including eosinophils and basophils [74]. Recently, chitin was shown to stimulate CCL2 secretion from airway epithelial cells and induce CCR2-dependent innate allergic inflammation in the lung [75]. Taken together, chitin could more likely play a role in the innate immunity activation by HDM but the precise mechanisms remain to be elucidated.

Although mammals do not produce chitin, acidic mammalian chitinase (AMCase) and the chitinase-like protein (CLP) are not only expressed in these organisms but upregulated in asthmatic patients [73]. Moreover, in an aeroallergen asthma model, AMCase was induced via a Th2-specific, IL-13-mediated pathway in epithelial cells and macrophages [76]. However, the inhibition of the chitinolytic activity through *in vivo* AMCase inhibitor administration as well as the modulation of AMCase gene expression by using AMCase transgenic or gene deficient mice had no real impact on the severity of the airway inflammation [77]. At least, HDM group allergen 15 and 18 displayed sequence homology with chitinases [27]. However, their contribution in the HDM-induced innate immunity activation was not yet explored.

## 8. Role of NLR Activation in HDM Allergy

To date, the innate immunity activation by HDM through NRL pathways was poorly characterized. Partial and contrasting results were reported only for the role of NLRP3 inflammasome: a cytosolic multiprotein complex associating a NLR oligomer with the NLR adaptor protein PYCARD (ASC) and caspase-1 [78]. Once the inflammasome is formed following stimulation, caspase-1 becomes activated and cleaves pro-IL-1*β* and pro-IL-18 into their mature cytokines. Wild-type and NLRP3-deficient mice developed comparable allergic airway inflammation induced by HDM extracts suggesting that NLRP3 does not contribute to the HDM allergic response [79, 80]. However, the activation of human keratinocytes with HDM extracts triggered caspase-1 activation and promoted the caspase-dependent release of IL-1*β* and IL-18 suggesting the importance of the inflammasome engagement in the pathogenesis of AD [81]. The assembly of NLRP3 inflammasome through the HDM cysteine protease Der p 1 was clearly evidenced in those cells.

Uric acid crystals is a well-known DAMP released from stressed or necrotic cells which induced neutrophilic inflammation through engagement of the NLRP3 inflammasome and release of IL-1*β* [82, 83]. It was recently showed that intranasal HDM extracts administration leads to a drastic TLR4-dependent increase of uric acid concentration in BAL fluid and lungs [79]. Intratracheal administration of uricase at the time of sensitization strongly decreased the typical airway allergic inflammation. Surprisingly, the uric acid-mediated Th2 allergic response occurred independently from NLRP3, Pycard, MyD88, or IL-1R but resulted from DC activation through spleen tyrosine kinase and PI3-kinase *δ* signaling in a receptor-independent manner [79].

ATP and other nucleotides represent important dangerous signals which can also engage the NLRP3 inflammasome through the binding to the purinergic receptors (P2XR and P2YR). Although, to date, the increase of ATP in the airways was observed only using a ovalbumin-based experimental asthma model [84], the use of P2X7R−/− mice as well as a specific P2X_7_R-antagonist showed clearly the involvement of P2X7R signaling in HDM-triggered allergic asthma [85]. 

## 9. Protease-Dependent Innate Signaling in HDM Allergy

As described above, four HDM allergen groups present in the mite faeces display proteolytic activities: allergens from group 1 are cysteine proteases, whereas those from groups 3, 6, 9 are serine proteases. When the mite faeces reach the lower airways of the lung to release the allergens, the first target of proteolytically active allergens are the soluble antiprotease-based lung defenses. Group 1 HDM allergens indeed degraded airway *α*1-antitrypsin inhibitor [86], elastase-specific inhibitor (Elafin), and secretory leukocyte protease inhibitor [87] resulting in enhanced tissue damage and immune activation. In addition, group 1 HDM allergens was able to inactivate lung surfactant proteins A and D (collectins) [88], which are known to play not only significant roles in the innate defense mechanism against pathogens but also in the protection against allergen-induced airway inflammation [89].

As the airway epithelium is the first point of contact with airborne allergens, numerous studies evaluated the direct effect of the HDM proteolytic activities on this layer of cells and particularly at the level of the breakdown of epithelial barrier integrity. In this context, tight junction proteins, the most apical component of intercellular junctional complexes, regulate the epithelial barrier to prevent the passage of respiratory pathogens and toxins from the airway lumen into the systemic circulation. These proteins control also the passage of ions, water, and various macromolecules through paracellular spaces from the apical to basolateral surface [90]. Impaired the barrier function of the airway epithelium was commonly described in asthmatics patients [91]. The development of asthma can be partly a consequence of such epithelium disruption which facilitates the penetration into submucosal tissues of infectious particles/allergens to interact with immune and inflammatory cells. 

The direct effect of HDM protease allergen on airway epithelial cells was first published in 1995 [92], the cysteine protease activity of HDM extracts elicited significant increases in the permeability of isolated sheets of bronchial mucosa as judged by a progressive increase in bioelectrical conductance [93]. Although DCs can take up directly airway antigens through dendritic extension across the epithelial layer while maintaining barrier integrity by formation of tight junctions [94], mite proteases (Der p 1, 3, 6, and 9) [95, 96] can cleave tight junction proteins to gain access to DCs in subepithelial tissue through occludin and zonula occludens-1 (ZO-1) degradation.

However, very surprising results came from the effects of different HDM extracts varying deeply in proteolytic activities on epithelial barrier function, release of proinflammatory cytokines and induction of Th2 responses, *in vitro* and *in vivo* [37]. This new paper established the existence of causal relationships between airway epithelial responses and the induction of a Th2-polarised immune response. But, strikingly, the HDM extract which exerted the most pronounced effects was the one which displayed the lowest serine and cysteine protease activity. These data highlighted that serine/cysteine proteases from HDM extracts are not critically required for disruption of epithelial barrier function *in vitro* and innate immune response. Future studies will have to identify which specific HDM components are responsible for the effects on epithelial barrier function in relation to allergic sensitisation.

Proteinase-activated receptors (PARs) are receptors activated by proteolytic cleavage of their extracellular N-terminus by serine proteases to reveal a tethered ligand sequence capable of autoactivating the receptor. PARs, although not considered as authentic PRRs, participate to the innate immune defense and immune surveillance by sensing the extracellular microbial proteases [97]. PAR-2 has been associated with the inflammatory response to infection and microbial proteases. Moreover, a cooperativity in PAR-2 and TLR4 signal transduction was clearly demonstrated [98] and PAR-2 activation altered E-cadherin adhesion to increase the paracellular permeability [99]. In the context of asthma, stimulation of PAR-2 receptors in airways leads to increased water and chloride (Cl^−^) secretion due to elevated expression of   Ca^2+^-activate Cl^−^ channels [100]. 

PAR-2 activation by HDM extracts triggered glandular fluid hypersecretion in patients suffering from allergic rhinitis or chronic rhinosinusitis [101]. The PAR-2 dependent fluid secretion in HDM-treated human airway epithelia resulted from apical Cl^−^ movement via cystic fibrosis transmembrane conductance regulator (CFTR) and Ca^2+^-activated Cl^−^ channel [102]. Taken together, these data suggested that the protease activity of HDM plays a role in fluid hypersecretion of airway mucosa. 

Stimulation of airway epithelial cells with Der p 1 [57, 103–106], Der p 3 [106–108], and Der p 9 [104, 107] induced the protease-dependent secretion of large amounts of IL-6, IL-8, MCP-1, GM-CSF, RANTES, and Eotaxin. These proinflammatory, pro-Th2 cytokines as well as chemokines recruit and/or activate DC, Th2 cells as well as granulocytes (eosinophils, neutrophils and basophils). Whether this cell activation was shown to be at least mediated through PAR-2 signaling pathways for Der p 3 [106, 107] and Der p 9 [107], contradictory results were published on Der p 1. Although one previous study demonstrated the PAR-2-dependence in the Der p 1-induced cytokine secretion in human airway epithelial cells [105], recent data evidenced, by contrast, that Der p 1 inactivates PAR-2 through inadequate receptor proteolytic cleavages [57, 106, 108] suggesting that another protease-sensitive cell surface receptor(s) is (are) more likely the target(s) for the Der p 1-induced cytokine release in airway epithelial cells. A very recent study demonstrated that Der p 1 stimulated protease-dependent production of TSLP and pro-Th2 cytokine IL-25 in airway epithelial cells [109]. Although a PAR-2-dependent TSLP upregulation was recently evidenced in airway epithelium [110], it is unlikely that Der-p-1-induced TSLP results from PAR-2 activation according to the above results.

It must be also pointed out that the airway epithelial cell activation by proteolytically active Der p 1 stimulated the secretion of chemokines involved in selective dendritic cell recruitment as CCL2, CCL5, CCL20, and IP-10 [103].

However, it is interesting to note that PAR-1 and PAR-2 expression was upregulated in primary human nasal epithelial cells incubated with Der p 1 and that Der p 1 stimulated a PAR-2-dependent downregulation of connexin, a protein involved in the integrity of tight junctions [111, 112]. 

The specific proteolytic cleavages of CD40 and DC-SIGN by Der p 1 on the DCs cell surface resulted in a downregulation of the Th1 polarization or tolerance through the reduction of IL-12p70 and extracellular thiols production (CD40 cleavage) as well as a decline in binding to the naïve T cell DC-SIGN ligand ICAM-3 (DC-SIGN cleavage) [113–115]. The protease activity of Der p 1 was also responsible of the downregulation of IDO expression in DCs from HDM-sensitive subjects with asthma which can explain the loss of tolerance towards HDM allergens in these patients, the IDO expression being commonly associated with tolerogenic DCs [116]. 

Proteolytically active HDM extracts or Der p 1 triggered airway epithelial cells, skin keratinocytes, DCs, eosinophils, basophils, macrophages to secrete large amounts of proinflammatory, pro-Th2 cytokines as well as chemokines to recruit/activate DC, Th2 cells as well as granulocytes (eosinophils, neutrophils and basophils) including IL-1*β*, -4, -5, -6, -8, -9, -13, MCP-1, GM-CSF, TNF-*α*, RANTES, Eotaxin, CCL-2, -5, -20, IP-10, TARC, MDC [117]. However, the protease-sensitive target(s) remain(s) to be identified. Der p 1 was shown to induce in neutrophils a cysteine protease-dependent release of ROS, a common feature of asthmatic airway [118].

## 10. HDM-Promoted Airway Remodeling

Airway remodeling, which characterizes the structural changes to the airways in asthmatic patients, is a not fully elucidated complex process leading to the irreversible loss of lung function and airway hyperresponsiveness. Airway remodeling includes notably epithelial changes and loss of epithelial integrity, thickening of the basement membrane, mucus-gland and goblet-cell hyperplasia, smooth muscle hyperplasia, and increased airway vascularity [119]. The profibrotic cytokine TGF-*β* is one of the main mediators of tissue remodeling [120]. It is well accepted that epithelial-mesenchymal transition (EMT) is central to this long-term structural changes within the airways [121] and such phenotypic transition can be notably illustrated by downregulation of epithelial cell markers as ZO-1, occludin, E-cadherin together with expression of mesenchymal cell markers vimentin, and N-cadherin [119]. Two recent papers demonstrated that HDM proteases activate EMT in airway epithelial cells [122, 123]. HDM proteases induced E-cadherin internalization, enhanced *β*-catenin-dependent transcription, and downregulated cytokeratin in airway epithelia primed with TGF-*β* [122]. Inhibition of EGFR prevented HDM-induced EMT suggesting that dys-regulated EGFR signaling is critical in the airway remodeling process. Mite protease allergens induced redistribution of E-cadherin in human bronchial epithelial cells via EGFR-dependent activation of PAR-2 [123]. The presence of TGF-*β* prolonged the stimulation of EGFR signaling pathways. It is noteworthy that downregulation of E-cadherin results in EGFR-dependent upregulation of TARC and TSLP [124]. Taken together, these data clearly suggested that dys-regulated EGFR signaling induced by protease allergens is critical in the airway remodeling. It is interesting to note that HDM extracts could directly drive TGF-*β* expression from airway epithelial cells [125] and that Der f 1 could also cleave LAP latency-associated peptide (LAP) of TGF-*β* to activate latent TGF-*β* [126]. The PAR-2 activation by HDM extract in bronchial smooth muscle cells from asthmatics but not from healthy patients induced the release of IL-6, triggered the proliferation of these cells through downregulation of the transcription factor C/EBP*α* [127].

## 11. HDM Innate Immunity Activation through Unknown Cellular Targets

NF*κ*B-dependent upregulation of GM-CSF, IL-6, IL-8, MCP-1, and MIP-3*α* cytokines was elicited not only by Der p 2 but also by Der f 2, Eur m 2 amd Lep d 2 in bronchial epithelial cells [128, 129]. Der p 2 was able to stimulate *in vitro* as well as *in vivo* NGF and ROS production in airway epithelial through p38 and JNK MAPK signaling pathways. An antioxidant prevented the Der p 2-induced inflammation [130]. Recombinant Der f 2 can directly stimulate IL-13 production in the same target cells through a phospholipase D1-dependent pathway [131]. HDM extracts also promoted expression of cell surface c-KIT and its ligand, stem cell factor, on mouse DCs, resulting in sustained signaling downstream of KIT, upregulation of the Notch ligand Jagged-2, and finally IL-6 secretion [132]. 

## 12. IL-25, IL-33, TSLP: Key Regulators of HDM Allergy?

Whether great progress was made in understanding the mechanisms of molecular crosstalk between epithelial cells and immune cells, much attention has focused on the role of a triad of innate epithelial cell-derived cytokines: IL-25, IL-33, and TSLP. IL-25, IL-33 and TSLP are all implicated in promoting Th2-type immune responses and their individual contribution to the Th2 polarization have been mainly evaluated in deficient/transgenic mice, allergen sensitization mouse models or through systemic or airway administration of these cytokines [133]. 

TSLP conditions DCs to favor Th2 induction through prevention of IL-12 production and upregulation of the costimulatory molecule OX40L [134]. It must be pointed out that TSLP was overexpressed in bronchial biopsies from asthmatics and TSLP gene promoter polymorphisms were shown to be associated with susceptibility to bronchial asthma [135, 136]. TSLP induced the differentiation of basophils from bone marrow and basophils could represent the initial source of IL-4 to strengthen the Th2 polarization initiated by DCs [9, 137]. 

IL-33, which is normally sequestered in the nucleus of epithelial cells, can be secreted upon epithelial activation or injury. For that reason, IL-33 can be considered as an alarmin bridging tissue damage and immune response [138]. Higher IL-33 expression was observed in bronchial epithelium from asthmatics compared with that from healthy subjects [139]. *In vivo* IL-33 administration to mice triggered strong Th2 responses characterized by lung eosinophilia as well as increased levels of serum IgE and IgA [140]. This Th2 polarization was mediated through activation of lung DCs via the IL-33 binding to ST2 receptor [141]. IL-33 drives production of cytokines and chemokines by Th2 cells, mast cells, basophils, eosinophils, NKT cells, and NK cells [140].

IL-25 regulates multiple aspects of mucosal immunity by promoting Th2 inflammation via production of IL-4, IL-5, and IL-13 through notably the direct IL-25R signaling-dependent activation of Th2 cells. It must be pointed out that IL-25R was shown to be more expressed in Th2 than other effector Th cells [142, 143]. Overexpression of IL-25 in lung epithelial cells led to mucus production and airway infiltration of macrophages and eosinophils [144]. Patients with atopic airway disease have been reported to have elevated serum levels of IL-17A and IL-25 [145].

IL-25 and IL-33 are also strong stimulators of type 2 innate lymphoid cells (ILC2s) [14]. This family of (T, B, NK, monocyte, mast, basophil) negative ILC cells, producing large amount of IL-5 and IL-13, comprised natural helper cells, nuocytes, or multipotent progenitors. Interestingly, ILC2s accumulated into the lungs of mice infected influenza virus through IL-33 dependence and triggered airway hyperreactivity through IL-13 secretion [146]. Finally, ILC2s are commonly found in patients with chronic rhinitis [147].

Initially, three reports evidenced the epithelial production of IL-25, IL-33, and TSLP following HDM extracts administration into the airways of mice [47, 48, 79] and consequently underlined the role of this cytokine triad in the induction of HDM airway inflammation. A very recent research, complementing these previous studies, demonstrated for the first time that HDM-induced asthma was also accompanied by increased numbers of ILC2s in lung and BAL [148]. Moreover, in that HDM allergy model, pulmonary ILC2s produced large amount of IL-5 and IL-13. Remarkably, such ILC2-induced Th2 cytokine production was at the same range as found for the Th2 cells, highlighting the key role of ILC2s in the HDM-induced Th2 polarization. Finally, through the use of neutralizing antibodies or mice deficient in TSLP, IL-25, or IL-33 signaling, the HDM-induced TSLP and IL-25 production was shown to be dispensable for the airway sensitization of HDM. In contrast, IL-33 played an essential role for the HDM-induced Th2 immunity through OX40L on DCs and activation of ILC2s [149]. Although this study did not elucidate the mechanism by which HDM allergens stimulated the production of TSLP, IL-25, and IL-33 into the airways, it is tempting to speculate that the initiation of HDM allergy results from three main steps: IL-33 stimulation, OX40L upregulation, and expansion of ILC2s into the airways.

## 13. HDM Activation of Skin Innate Immunity

Atopic dermatitis (AD) is a common, chronic inflammatory skin disease characterized by immune abnormalities and a disturbed epidermal barrier, resulting in increased transepidermal water loss (TEWL) and permeation of allergens, irritants, and microbes [150]. The key role of filaggrin (FLG), a protein contained in the granular layer of the epidermis regulating the aggregation of keratin filaments, was evidenced in atopic dermatitis as several loss-of-function mutations in *FLG* gene or FLG deficiency contribute to epidermal barrier dysfunction and was strongly associated with AD [151].

Alterations of innate immunity have been described in AD which trigger and aggravate the course of the disease. Notably, a defect of TLR2 expression or signaling was associated with AD. Single-nucleotide polymorphisms (SNPs) in the TLR2 gene as well as in genes encoding NOD1, NALP12 (another member of the NOD-leucine rich repeat-containing protein and NOD2 were associated with AD) [152].

The progression of atopic dermatitis to asthma and allergic rhinitis during the first several years of life is a phenomenon known as “atopic march” [153, 154]. A majority of patients with AD developed sensitizations to common inhalant allergens and the sensitization level was associated with the severity of AD as well as the risk of developing asthma [154]. Although the mechanism explaining the atopic march remains to be fully elucidated, it is evident that the direct contact of skin with allergens could trigger signals to initiate Th2 allergic response. 

As the major mite allergens Der p 1 and Der f 1 were detected on the surface of human skin [155], it is not surprising that HDM allergens play also a role in the development of atopic dermatitis [150, 156, 157]. HDM protease allergens weakened not only the barrier function of the skin [158] but impaired the epidermal permeability barrier recovery in both human and murine skin [159]. Repeated topical applications of HDM extracts in NC/Nga mice, a sensitive animal model for atopic dermatitis, triggered strong skin inflammatory responses through inflammatory cell infiltrates in the upper dermis, elevated specific IgE concentrations and CCL20, TARC and eotaxins upregulation [160]. HDM extracts as well as purified Der p 1 can directly stimulate epidermal keratinocytes or human skin equivalent to promote the secretion of IL-1*α*, TGF-*α*, GM-CSF, M-CSF, IL-6, IL-8, MCP-1, and TNF-*α* [161–164]. The PAR-2 activation by HDM serine proteases in keratinocytes elicited GM-CSF and IL-8 production [108]. PAR-2 was shown to regulate the epidermal permeability barrier function homeostasis and leukocyte recruitment to inflammatory sites within the skin [165]. 

Moreover, microbial compounds from HDM environment can directly stimulate skin innate immunity. First of all, LPS stimulated the production of various cytokines and chemokines including IL-1*β*, TNF-*α*, IL-8, GM-CSF, MIP-1*α*, and TARC in keratinocytes [166–169]. The treatment of primary keratinocytes by TLR3, TLR2-6, or TLR5 ligands as well as by atopic cytokine milieu (TNF-*α* + IL-4 + IL-13) lead to TSLP production [170]. Chitin as well as *β*-glucans modulated keratinocyte innate immunity through the production of IL-1*β*, IL-6, IL-8, or TSLP [171, 172].

A very recent study brought the experimental proof that TSLP makes the connection between atopic dermatitis and HDM-induced airway inflammation and, consequently, that this cytokine represents the key factor of the atopic march [173]. Indeed, TSLP was critical to promote skin allergic inflammation, allergen-induced Th2 response, allergen sensitization subsequently leading to allergic asthma. Moreover, the controlled production of TSLP in AD-skin (skin treated topically with MC903, a vitamin D3 analog) clearly indicated that TSLP concentration was correlated with the level of skin inflammation and the severity of HDM airway inflammation. The direct application of HDM extracts in skin of AD patients with histories of HDM allergy induced IL-33 and ST2 expressions suggesting a key role for IL-33-ST2 interaction in the pathogenesis of atopic dermatitis [174].

## 14. Is the HDM-Induced Innate Immunity in Healthy and Atopic Subjects Comparable? 

The present extensive paper clearly evidenced the key contribution of the innate immunity activation in the development of HDM allergy. Although the concentration of HDM allergens as well as contaminating LPS/*β*-glucans in the house dust can differ with the environment, we can consider that non atopic subjects, similar to atopic patients, can be potentially exposed to equal amounts of HDM allergens and microbial adjuvant compounds. Consequently, although we can speculate that innate immune signaling pathways can be similarly triggered in healthy individuals, such responses do not lead to the allergic sensitization suggesting qualitative and quantitative differences in HDM-induced innate immune responses between healthy and atopic subjects in terms of pathways, intensity, or timing. Numerous investigations reported the dysregulation of the innate immune system in atopic patients arising notably from genetic alterations of the skin and airway structural cells together with mutations and/or modulation of PRRs, PRR-associated signaling molecules or difference in basal expression of key pro-Th2 molecules [175, 176]. 

The airway epithelium from atopic patients was abnormal. Indeed, bronchial biopsies from asthmatic subjects displayed disruption of tight junctions confirming that bronchial epithelial barrier in asthma is compromised [177]. The expression of EGFR, E-cadherin, ZO-1 was dysregulated in asthmatics airway epithelia together with aberrant expression proinflammatory transcription factors including the STAT pathways [178–180].

At steady state, a larger proportion of DCs are observed in the airway mucosa of patients with allergic asthma compared with non atopic donors, and the density of this network was also upregulated after allergen exposure [181]. Bronchial epithelial cells from allergic asthmatics display an increase in the baseline and Der-p-1-induced expression of proinflammatory cytokines/chemokines compared with non atopics [103, 182]. HDM extracts stimulated thrombomodulin (CD141) upregulation in DCs from atopics but a CD141 downregulation in stimulated DCs from non atopic subjects [182]. Moreover, CD141+DCs elicited a more pronounced Th2-bias response than CD141-DCs. Whereas normal skin and airway epithelium were reported to express undetectable and low amount of TSLP, respectively, TSLP was overexpressed at the lesional sites of AD and in the bronchial epithelium and submucosa in asthmatics [135, 183]. A TSLP gene variant was shown to be associated with asthma and airway hyperresponsiveness [136]. Bronchial epithelium of asthmatics expressed elevated levels of IL-33 compared with that isolated from healthy controls, which strengthen the concept that bronchial epithelial cells in asthma exhibit altered and proinflammatory phenotype compared with cells from healthy controls [139].

Moreover, the upregulation of PAR-2 together with a prolonged airway epithelial NF*κ*B activation in bronchial biopsies from allergic patients compared with nonatopics [184, 185]. 

Increased TLR signaling was commonly associated with several pulmonary diseases and there is growing evidence that asthma and atopy are positively associated with polymorphisms in genes coding for TLR2, TLR4, TLR1, and TLR6 as well as TLR-related MyD88-dependent pathway molecules [186]. Differences in epigenetic modifications, such as DNA methylation, were detected in airway epithelia from healthy and asthmatic subjects and could contribute to the modulation of the innate immunity triggered by HDM [187]. 

Taken together, these data highlighted large abnormalities of the innate immune system between atopic patients and healthy subjects and particularly at the level of the airway epithelium. Consequently, we can consider that, in contact with the same dose of HDM allergens and/or microbial adjuvants, atopic subjects compared with healthy individuals will develop exacerbated innate immunity responses which could reach a threshold limit to trigger and/or sustain the allergic response.

## 15. Concluding Remarks

**Figure 2:**
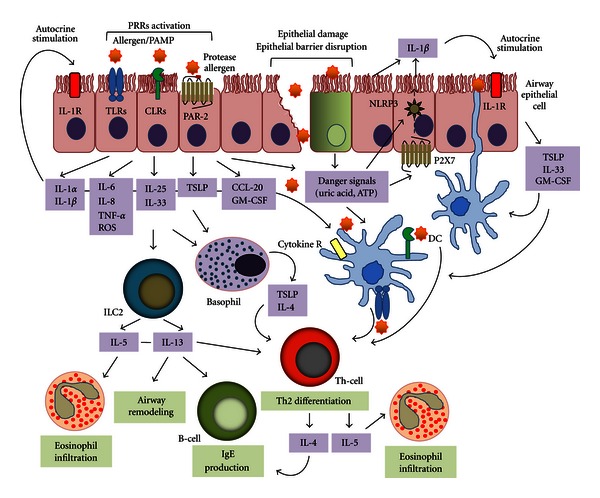
Simplified model of the HDM-induced innate immune activation leading to allergic asthma. The induction of Th2 immunity by HDM allergens results from the stimulations of different innate immune pathways. HDM protease allergens specifically cleave protease-sensitive receptors including PAR-2, disrupt epithelial barrier to gain access to DCs, and cause tissue injuries to release DAMPs such as ATP and uric acid. Contaminating microbial PAMPs associated or not with lipid-binding allergens trigger numerous PRRs which can produce also DAMPs whereas HDM glycan activation of DCs is mediated through CLR ligation. These signaling pathways result in the massive upregulation of innate cytokines/chemokines as IL-1*β*, IL-6, TSLP, IL-25, IL-33, GM-CSF, or CCL20 to recruit and activate inflammatory cells and to induce Th2 differentiation. Notably, TSLP mediates OX40L and IL-4 expression in DCs and basophils, respectively, to initiate Th2-polarized response. IL-25 as well as IL-33 are strong activators of ILC2s which, similar to allergen-specific Th2 cells, provide large amount of the Th2 cytokines IL-5 and IL-13 to generate the main features of the allergic airway inflammation such as IgE secretion by B cells, eosinophil recruitment, and airway remodeling.

Within the last decade, substantial advances have been made in identifying the role of innate immunity activation by HDM allergens in the initiation and maintenance of the allergen-specific Th2-biased adaptive responses. HDM can be definitively considered as a complex organism not only carrying allergens with intrinsic biological properties but also containing endosymbiotic and/or contaminating microbial adjuvants. These allergen/adjuvants “formulations” efficiently match together to stimulate the innate immune pathways in mucosal surfaces, DCs and in inflammatory cells. The innate immune activation in HDM allergy can be summarized in Figure 2.

To date, more than twenty different HDM allergens were identified, according to their IgE reactivity and it is plausible, thanks to the use of more sensitive technologies, that new HDM allergens will be identified in the near future. According to their allergenicity based on strict IgE reactivities, HDM allergens were roughly classified into major and minor allergenic proteins. IgE binding assays with sera from HDM allergic subjects worldwide evidenced that group 1 and group 2 HDM allergens contribute to more than 50% of the total allergen-specific IgE response, whereas 30% of specific IgE are equally addressed to groups 4, 5, and 7. The prevalence of the IgE reactivity to other HDM allergens was weak [23]. But the present paper strikingly suggests that this old-fashioned HDM allergen hierarchy must be replaced by a classification based on their capacity to stimulate innate immune responses. In this context, at least two allergen families could play a critical role in the HDM-induced innate immunity: the proteases represented by groups 1, 3, 6, and 9 which directly trigger signaling through proteolytic attacks and the lipid-binding proteins including groups 2, 5, 7, 13, 14, and 21 which could transport microbial lipid-based PAMPs. It must be pointed out that, whether the triggering of innate immunity was demonstrated for groups 1, 3, 9, and 2, such stimulations remain to be evidenced for the other groups. Other key questions are to know whether these lipid-transfer proteins display intrinsic allergenicity alone or strictly through the association with their lipid cargo. Whereas the proteolytic activities of group 1 HDM allergens largely contribute to the stimulation of the innate immunity, particular notice must be paid to the identification of cell surface Der p 1 targets and especially on the airway epithelium.

Remarkably, it appeared that microbial adjuvant compounds detected in HDM carriers participate largely to the HDM-induced Th2 polarization through PRRs activation. Several important questions remain unanswered such as the full identification of the microbial compounds present in HDM extracts/environment as well as the receptors and the signaling pathways engaged by such compounds. Whether HDM allergens, in the absence of any trace of microbial compounds can still develop allergic response is an interesting hypothesis to be tested.

Although the in-depth characterization of the allergenic determinants of mite allergens in regards with their capacity to trigger innate immune signaling pathways needs more progress, the data outlined in this present paper have a potentially important implication for human health. Particularly, the design of new drugs that selectively inhibit crucial aspects of the epithelial-immune interaction could potentially inhibit innate immune signaling driving sustained allergic inflammation. Therapeutic targeting of the epithelium-derived cytokines as TSLP, GM-CSF, IL-25, IL-33, IL-1, or PAR-2 could reduce the risk of HDM-induced allergic sensitization.
